# A modified perturb and observe MPPT algorithm for PEMFC with rapid convergence and low power oscillation

**DOI:** 10.1038/s41598-025-09947-3

**Published:** 2025-07-30

**Authors:** Resat Celikel, Omur Aydogmus, Musa Yilmaz

**Affiliations:** 1 Department of Mechatronics Engineering, Firat University, 23200 Elazig, Turkey; 2 Department of Electrical and Electronics Engineering, Batman University, Batman, 72100 Turkey; 3 Bourns College of Engineering, Center for Environmental Research and Technology, University of California at Riverside, Riverside, CA 92521 USA

**Keywords:** Fuell cell, PEMFC, MPPT, Perturb &observe, Optimization algorithm, Energy science and technology, Engineering

## Abstract

Proton Exchange Membrane Fuel Cells (PEMFCs) enable continuous energy production regardless of environmental conditions due to the storability of hydrogen. When examining the current–power (I–P) curve of a PEMFC under steady-state operating conditions, maximum power is observed at a specific current level. To extract this power, Maximum Power Point Tracking (MPPT) algorithms are employed. These algorithms should feature a simple structure and rapidly track the maximum power point. However, intelligent and optimization-based methods in the literature often involve high computational complexity. In this study, a modified Perturb and Observe (P&O)-based MPPT algorithm is developed to achieve a fast steady-state response under varying PEMFC operating conditions. The proposed algorithm also minimizes power oscillations in the steady state. Its performance is evaluated in a MATLAB/Simulink environment under five different scenarios. A comparative analysis is conducted against the conventional P&O and optimization-based MPPT algorithms, including Particle Swarm Optimization (PSO), Cuckoo Search Algorithm (CSA), and Genetic Algorithm (GA). The results, presented graphically, demonstrate the advantages of the proposed approach.

## Introduction

As technology advances and the global population increases, energy demand continues to rise. While fossil fuels remain a significant energy source, their depletion and the environmental issues associated with their use have become critical concerns. Consequently, the need for renewable energy sources has grown in recent years. Solar, wind, and wave energy are among the most widely used renewable energy sources; however, the energy they produce is highly dependent on fluctuating atmospheric conditions^1–3^. Ensuring continuity in energy production remains a major challenge in systems based on these sources. In recent years, Fuel Cells (FCs) have attracted significant attention due to their low emissions and high efficiency^4^. FCs are categorized into six types based on their operating principles. Among these, Proton Exchange Membrane Fuel Cells (PEMFCs) stand out for their low operating temperatures, fast startup, and high power density^5^. A PEMFC generates direct current (DC) electricity and water as long as fuel (e.g., hydrogen) and an oxidant (e.g., oxygen) are supplied^6^. The ability to store hydrogen enables uninterrupted power flow, allowing PEMFCs to operate independently of atmospheric conditions. When examining the current–voltage (I–V) curve of a PEMFC under steady-state conditions, a point can be identified where the maximum power is produced. To operate at this point, a DC–DC converter is used in conjunction with a Maximum Power Point Tracking (MPPT) algorithm that adjusts the converter’s switching to extract the maximum available power^7^.

Among MPPT techniques, Perturb and Observe (P&O) is one of the oldest and most widely used due to its simplicity, tunable parameters, and ease of implementation. However, it suffers from drawbacks such as delayed responses during transients and power oscillations in steady-state operation^8–10^. To address these issues, various modified P&O algorithms have been proposed, aiming to reduce steady-state power fluctuations and enhance performance under dynamic conditions^11–13^. Another widely adopted method is Incremental Conductance (InC), which can be implemented with fixed, variable, or fuzzy step sizes^14,15^.

Beyond traditional techniques, numerous optimization-based MPPT algorithms have been introduced. These include Particle Swarm Optimization (PSO)-based PID control^16^, Salp Swarm Algorithm^17^, Whale Optimization Algorithm^18^, Jaya Optimization^19^, Ingenious Golden Section Search^20^, Squirrel Search Optimization, and the Cuckoo Search Algorithm^21^. Artificial intelligence-based approaches have also gained traction. For instance, a Radial Basis Function Network-based MPPT algorithm has been developed for grid-connected systems and compared with InC and fuzzy logic-based approaches^22^. Similarly, a Unified Firefly Ersatz Neural Network-based MPPT method has been applied in fuel-cell-powered electric vehicles, showing competitive performance against PSO and Grey Wolf Optimization (GWO) algorithms^23^. Other studies have proposed ANFIS-based MPPT for BLDC-motor-driven electric vehicles^24^, as well as hybrid controllers combining artificial neural networks (ANN) with type-2 fuzzy logic for power quality enhancement in electrical systems^25^. Machine learning techniques have also been explored for high-efficiency MPPT in PEMFC systems^26,27^. In addition to AI-based strategies, fuzzy logic (FL)-based MPPT methods are also widely used. A high-efficiency FL-based MPPT algorithm using the Equilibrium Optimizer has been implemented^28^, and a Modified Fluid Search Optimization algorithm has been employed to fine-tune FL parameters in grid-connected PEMFC systems^29^. Recently, Type-3 FL algorithms have demonstrated strong performance in control applications^30^. The Bald Eagle Search method has been used to optimize Type-3 FL parameters for adaptive MPPT control^31^. Moreover, an improved Beta-Fuzzy Logic-based MPPT algorithm has been integrated into a PEMFC system with a novel DC–DC converter design^32^. Based on a comprehensive literature review, MPPT algorithms for PEMFCs can be categorized into three main groups: (1) traditional methods such as P&O and InC, which are simple and cost-effective but suffer from performance limitations; (2) optimization-based techniques, which offer improved performance but often require significant computational resources, making real-time implementation challenging; and (3) artificial intelligence and fuzzy logic-based approaches, which provide high accuracy and adaptability but come with increased complexity and training data requirements.

In this study, a modified P&O (M-P&O)-based MPPT method is developed to rapidly reach the maximum power point with improved performance during startup and reduced steady-state power oscillations. The proposed method applies a sequential duty cycle strategy to the DC–DC converter during the initial startup phase to perform a power scan. Based on the measured power values, the appropriate duty cycle range is determined. As the system nears the maximum power point, it transitions to a second phase governed by a modified P&O structure for fine-tuned control.

In the traditional P&O method, if the duty cycle increment is large, the system reaches the steady state more quickly; however, this leads to significant power fluctuations in steady-state operation. Conversely, when the increment is small, the steady state is achieved more slowly, but power fluctuations are reduced. Completely eliminating power fluctuations in the steady state requires a very small duty cycle increment, which significantly degrades both the convergence time and the transient performance during power changes. A critical limitation of ANN-based MPPT methods is the dependence on training datasets. Although these datasets vary across different systems, the accuracy of the data is crucial to the overall performance of the algorithm. More effective MPPT performance can be achieved with highly accurate datasets. In contrast, the proposed method does not require any training data, unlike ANN- or ANFIS-based MPPT techniques. Moreover, artificial intelligence-based algorithms are generally difficult to implement and require considerable computational resources. Optimization techniques are also commonly used to determine PEMFC parameters and extract maximum power^33,34^. While traditional algorithms such as PSO, CSA, and GA are widely employed, more advanced methods have also been developed. However, their complex structures often hinder practical implementation. The proposed algorithm offers a significant advantage by outperforming these methods in terms of both simplicity and performance. Using the proposed approach, the system rapidly achieved the maximum power level upon startup. In steady-state conditions, power fluctuations were minimized to negligible levels. Since the maximum power output of a PEMFC varies with changes in temperature and average water activity, the ability of the proposed algorithm to quickly adapt and track these changes is particularly valuable. The proposed algorithm is distinguished not only by its high performance but also by its ease of implementation, making it highly advantageous for practical applications.

To evaluate the effectiveness of the proposed method, a PEMFC system is simulated in MATLAB/Simulink under five different operational scenarios. The performance of the proposed algorithm is compared with that of the traditional P&O method and optimization-based algorithms, including PSO, CSA, and GA. In each scenario, the proposed method demonstrates superior performance in both transient and steady-state conditions. Graphical results highlight the advantages of the proposed approach, including its simple structure, significantly reduced power fluctuations during steady-state operation, and its rapid convergence to the new maximum power point following changes in operating conditions.

## Dynamic model of PEMFC

**Fig. 1:**
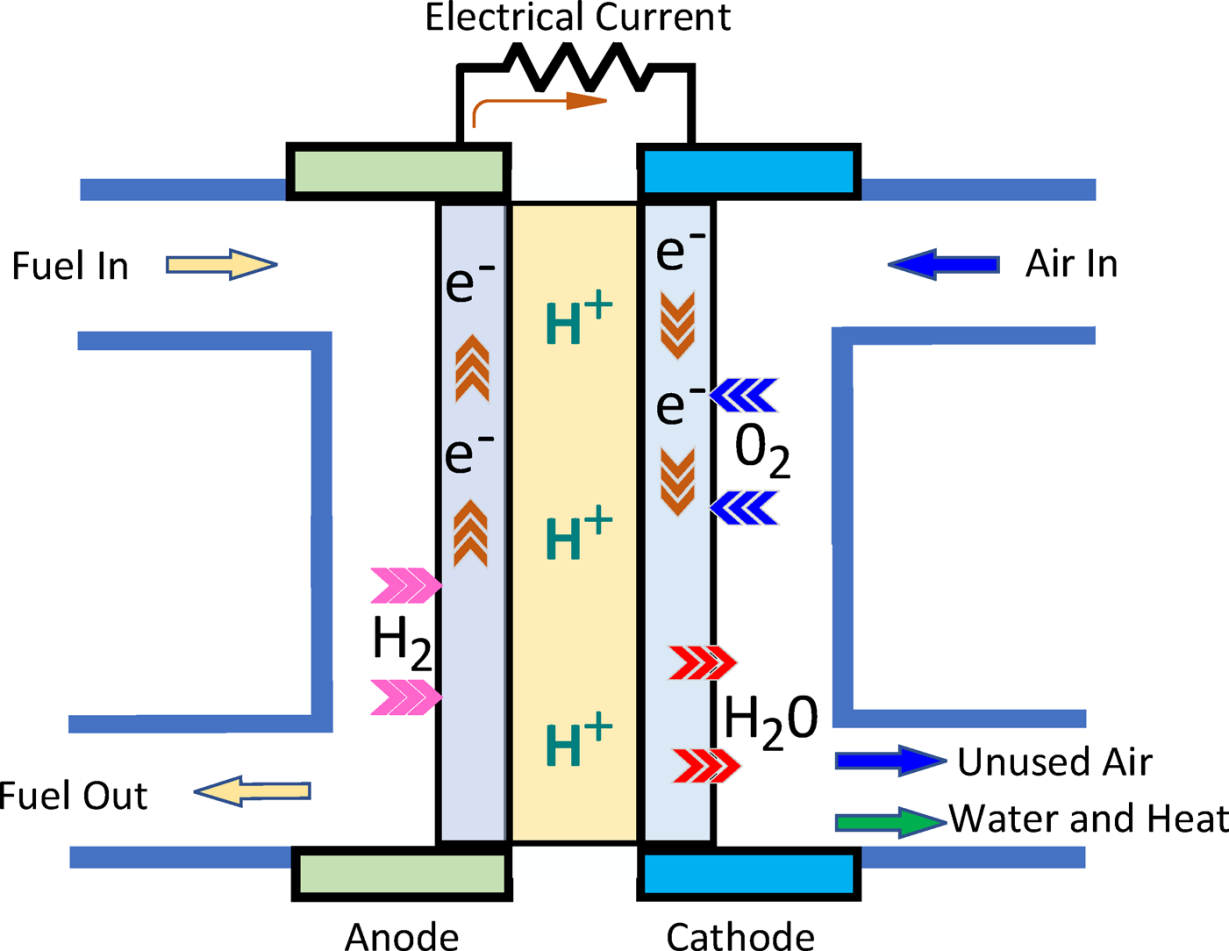
Structure of the PEMCF system.

A PEMFC is an energy conversion device that releases energy in the form of electricity and heat by utilizing the catalytic oxidation of hydrogen at the anode and the reduction of oxygen at the cathode, with water as a non-polluting byproduct. At the anode, a catalyst fuel, typically hydrogen, undergoes oxidation, converting the fuel into positively charged ions and negatively charged electrons. The electrolyte is a material specifically designed to allow ions to pass through but prevent electrons from doing so. The free electrons flow through a conductor, generating an electric current. The ions then pass through the electrolyte to the cathode, where they combine with electrons. Subsequently, the ions and electrons react with a third chemical, typically oxygen, producing water and heat. The typical operation of a PEMFC is illustrated in Fig. 1. A PEMFC can be fully described using two models: one for gas transport and the other for polarization curves.

### Dynamic gas transport model

The relationship between the gas flow through a valve and the partial pressure is given by Eq. (1) and Eq. (2).

1 $$\:\frac{{q}_{{H}_{2}}}{{P}_{{H}_{2}}}=\frac{{\mathcal{k}}_{an}}{\sqrt{{M}_{H2}}}={\mathcal{k}}_{H2}$$

2 $$\:\frac{{q}_{{O}_{2}}}{{P}_{{O}_{2}}}=\frac{{\mathcal{k}}_{an}}{\sqrt{{M}_{O2}}}={\mathcal{k}}_{O2}.$$

Here, $\:{q}_{{H}_{2}}$ and $\:{q}_{{O}_{2}}$ are the molar flow rates (kmol s^− 1^), $\:{P}_{{H}_{2}}$and $\:{P}_{{O}_{2}}$ are the partial pressures (atm), $\:{\mathcal{k}}_{H2}$ and $\:{\mathcal{k}}_{O2}$ are the valve molar constants (kmol (atm s)^−1^), and $\:{M}_{H2}$ and $\:{M}_{O2}$ are the molar masses of hydrogen and oxygen, respectively. $\:{\mathcal{k}}_{an}$ is an anode valve constant ($\:\sqrt{kmol\:kg\:{\left(atm\:s\right)}^{-1}}$). The partial pressure derivative of hydrogen can be calculated as shown in Eq. (3).

3 $$\:\frac{d}{dt}{P}_{{H}_{2}}=\frac{RT}{{V}_{an}}\left({q}_{{H}_{2}}^{in}+{q}_{{H}_{2}}^{out}+{q}_{{H}_{2}}^{r}\right).$$

Here, is the gas constant (J (kmol . K)^− 1^), *T* is the absolute temperature (K), $\:{V}_{an}$ is the anode volume, $\:{q}_{{H}_{2}}^{in}\:$is the hydrogen inlet flow (kmol s^−1^), $\:{q}_{{H}_{2}}^{out}\:\:$is the hydrogen outlet flow (kmol s − 1), and $\:{q}_{{H}_{2}}^{r}\:\:\:$is the hydrogen flow entering the reaction (kmol s^−1^). The amount of hydrogen consumed in the reaction can be calculated as shown in Eq. (4).

4 $$\:{q}_{{H}_{2}}^{r}=\frac{{N}_{FC}{I}_{FC}}{2F}=2{k}_{r}{I}_{FC}.$$

Here, $\:{N}_{FC}$ is the number of series-wound PEMFCs, $\:{I}_{FC}$ is the current of the PEMFC (A), $\:F$ is the Faraday constant (C kmol^− 1^), and $\:{k}_{r}\:\:$is the model constant (kmol (sA)^−1^). When Eq. 2 is rewritten and the equation given in Eq. (3) is solved, $\:{P}_{{H}_{2}}$ can be written as shown in Eq. (5).

5 $$\:{P}_{{H}_{2}}\left(t\right)=\frac{1}{{k}_{{H}_{2}}}\left(2{k}_{r}{I}_{FC}{e}^{\left(\frac{-t}{{\tau\:}_{{H}_{2}}}\right)}+{q}_{{H}_{2}}^{in}-2{k}_{r}{I}_{FC}\right).$$

where,

6 $$\:{\tau\:}_{{H}_{2}}=\frac{{V}_{an}}{{k}_{{H}_{2}}RT}.$$

Similarly, the partial pressure of oxygen can be written as shown in Eq. (7).

7 $$\:{P}_{{O}_{2}}\left(t\right)=\frac{1}{{k}_{{O}_{2}}}\left(2{k}_{r}{I}_{FC}{e}^{\left(\frac{-t}{{\tau\:}_{{O}_{2}}}\right)}+{q}_{{O}_{2}}^{in}-2{k}_{r}{I}_{FC}\right).$$

where,

8 $$\:{\tau\:}_{{O}_{2}}=\frac{{V}_{an}}{{k}_{{O}_{2}}RT}.$$

### Polarization curve model

The voltage obtained from the outputs of the PEMFCs varies due to factors such as temperature, partial oxygen pressure, partial hydrogen pressure, and membrane water content. The voltage of a cell can be obtained as shown in Eq. (9)^35^.

9 $$\:{V}_{cell}={E}_{nerst}+{V}_{act}-{V}_{ohmic}-{V}_{con}.$$

Here, $\:{E}_{nerst}$, $\:{V}_{act}$, $\:{V}_{ohmic}$ ve $\:{V}_{con}$ represent the thermodynamic equilibrium voltage, activation voltage losses, ohmic voltage loss, and concentration voltage loss, respectively. The $\:{E}_{nerst}$ potential is given in Eq. (10).

10 $$\:{E}_{nerst}=1.229-8.5\times\:{10}^{-4}\left(T-298.15\right)+4.308\times\:{10}^{-5}T\left(ln{P}_{{H}_{2}}+0.5ln{P}_{{O}_{2}}\right).$$

Here,* T *represents the operating temperature of the PEMFC. The expression for $\:{V}_{act}\:$ is given in Eq. (11).

11 $$\:{V}_{act}={\xi\:}_{1}+{\xi\:}_{2}T+{\xi\:}_{3}Tln{C}_{{O}_{2}}+{\xi\:}_{4}Tln{I}_{FC}.$$

Here, $\:({\xi\:}_{1},\:{\xi\:}_{2},\:{\xi\:}_{3},\:{\xi\:}_{4})$ represent the parametric coefficients of the cell model, and $\:{C}_{{O}_{2}}$ represents the oxygen concentration, as seen in Eq. (12). The $\:{V}_{ohmic}$ is given in Eq. (13).

12 $$\:{C}_{{O}_{2}}=\frac{{P}_{{O}_{2}}}{(5.08\times\:{10}^{6})\times\:{e}^{-\frac{498}{T}}}$$

13 $$\:{V}_{ohmic}={I}_{FC}\times\:\frac{{r}_{M}{t}_{M}}{A}.$$

where, $\:{r}_{M}$ is the membrane resistance against proton conductivity (cm), $\:{t}_{M}$ is the membrane thickness (cm), and *A* is the active area of the cell (cm^2^). The membrane resistance is calculated as shown in Eq. (14)^36^.

14 $$\:{r}_{M}=\frac{181.6\:\left[1+0.03\left(\frac{{I}_{FC}}{A}\right)+0.0062{\left(\frac{T}{303}\right)}^{2}{\left(\frac{{I}_{FC}}{A}\right)}^{2.5}\right]}{\left[{\lambda\:}_{m}0.0634-3\left(\frac{{I}_{FC}}{A}\right){e}^{4.18\left(\frac{T-303}{T}\right)}\right]}.$$

The membrane resistance depends on $\:{\lambda\:}_{m}$ and the temperature *T.* $\:{\lambda\:}_{m}$, defined as the average water activity, is calculated as:

15 $$\:{\lambda\:}_{m}=\left\{\begin{array}{c}0.043+17.81{a}_{m}-39.85{a}_{m}^{2}+36{a}_{m}^{3},\:\:\:\:\:\:\:\:0<{a}_{m}<1\\\:14+1.4\left({a}_{m}-1\right)\:\:\:\:\:\:\:\:\:\:\:\:\:\:\:\:\:\:\:\:\:\:\:\:\:\:\:\:\:\:\:\:\:\:\:\:\:,\:\:\:\:\:\:\:\:\:1<{a}_{m}\le\:3\end{array}\right..$$

As seen in Eq. (16), $\:{a}_{m}$ is a function of the partial pressures of the anode water vapor $\:{P}_{v,\:an}\:$and the cathode water vapor$\:\:{P}_{v,\:ca}$. If case 1 occurs, $\:{a}_{m}$ must be between 0 and 1; otherwise, it must be between 1 and 3.

16 $$\:{a}_{m}=\frac{1}{2}\left({a}_{an}+{a}_{ca}\right)=\frac{1}{2}\left(\frac{{P}_{v,\:an}+{P}_{v,\:ca}}{{P}_{sat}}\right).$$

The saturation pressure $\:{P}_{sat}$ is expressed using the empirical formula in Eq. (17).

17 $$\:{P}_{sat}=-2.1794+0.02953T-9.1813\times\:{10}^{-5}{T}^{2}+1.4454\times\:{10}^{-5}{T}^{3}.$$

The high value of $\:{\lambda\:}_{m}$, which results from the oversaturation conditions, can reach up to 23. In this case, the concentration overvoltage can be calculated as shown in Eq. (18).

18 $$\:{V}_{con}=-\frac{RT}{nF}\times\:ln\left(1-\frac{{I}_{FC}}{{i}_{L}A}\right).$$

Here, *n* represents the number of electrons involved in the reaction, and $\:{i}_{L}$ indicates the limiting current. For a PEMFC system consisting of cells connected in a simple series configuration, the output voltage and power are defined as shown in Eqs. (19) and (20).

19 $$\:{V}_{FC}={N}_{FC}\times\:{V}_{cell}$$

20 $$\:{P}_{FC}={V}_{FC}\times\:{I}_{FC}.$$

## Proposed MPPT method

MPPT methods used in PEMFC systems can be developed using various techniques. While optimization and artificial intelligence-based methods offer superior performance, the challenges in implementation and complex algorithms present significant disadvantages. Simpler, high-performance algorithms, when applied, can also reduce the initial setup cost of the system.

**Fig. 2:**
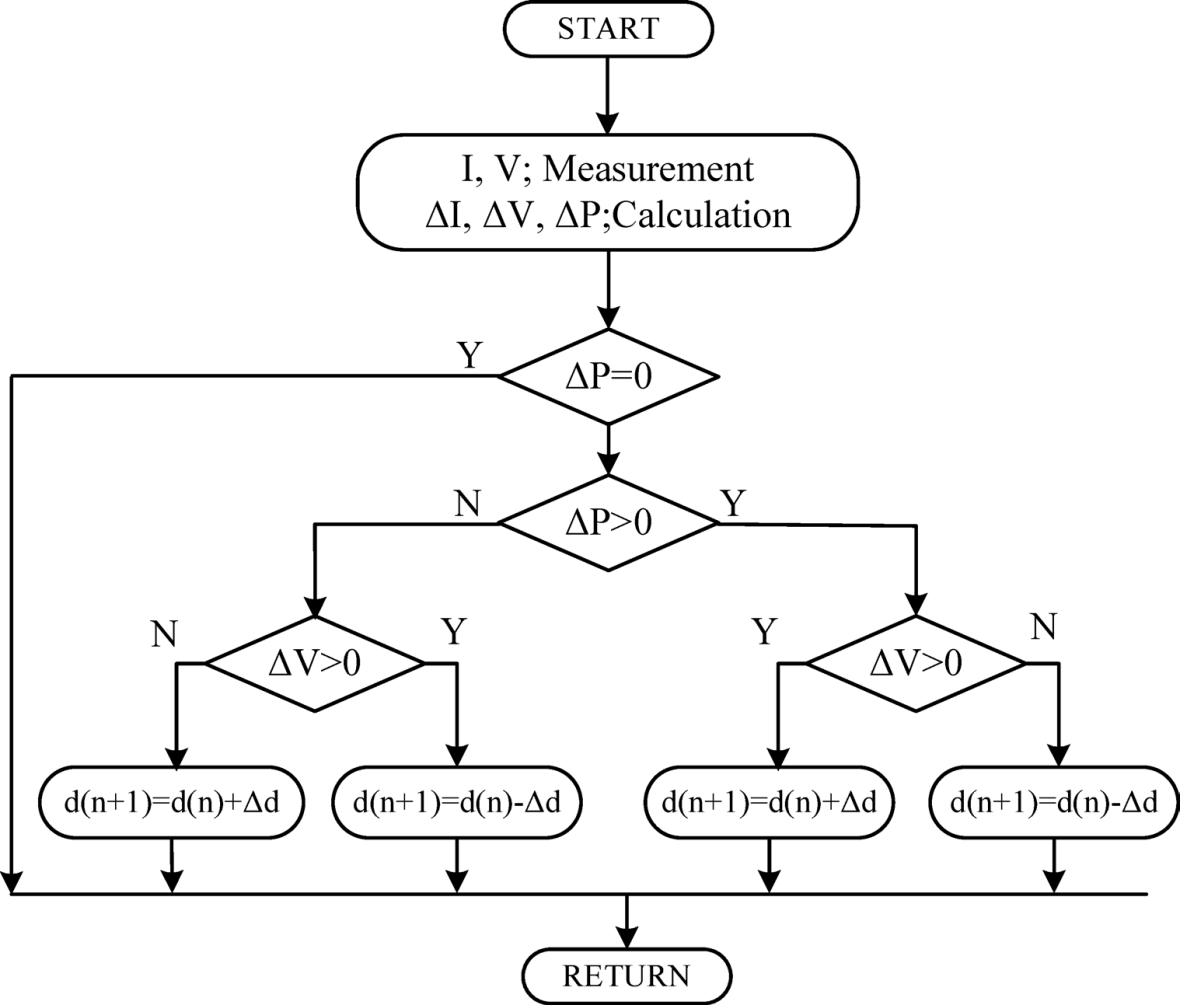
Flowchart of conventional P&O algorithm.

The P&O method shown in Fig. 2 is a simple MPPT method but has an important disadvantage. In this method, current and voltage values are measured, and changes in power and voltage are calculated. The value of the duty cycle applied to the boost converter is increased or decreased based on the positive or negative changes in power and voltage. The increment of the duty cycle is predetermined. When this value is large, high power fluctuations occur in the steady state. When the increment of the duty cycle is small, the time to reach the steady state is delayed.

In this study, a modified P&O algorithm has been developed. The proposed algorithm consists of two stages. In the first stage, the disadvantage of the P&O algorithm, which is the time to reach the steady state, has been significantly reduced. Initially, four tasks with equal spacing and fixed time intervals are applied sequentially over four periods. When the power change $\:\varDelta\:P>0.01$ condition is met, a counter performs counting, and each time the counter increases, a new duty cycle sequence is applied. During duty cycle transitions, power values are sampled. At the highest power value, the previous duty cycle is transferred as the starting duty cycle for the second stage. In the second stage, the starting duty cycle is increased by a certain ratio. The increase amount can be chosen randomly; however, in this study, it has been calculated as shown in Eq. (21) to ensure a balanced power distribution. The increase amount should be much smaller than the one in the previous section, ensuring convergence toward the maximum power.

21 $$\:{k}_{n}=\left.\frac{{D}_{n+1}-{D}_{n}-0.1}{{4}^{n}}\right\}\:if\:0<k\le\:3.$$

**Fig. 3:**
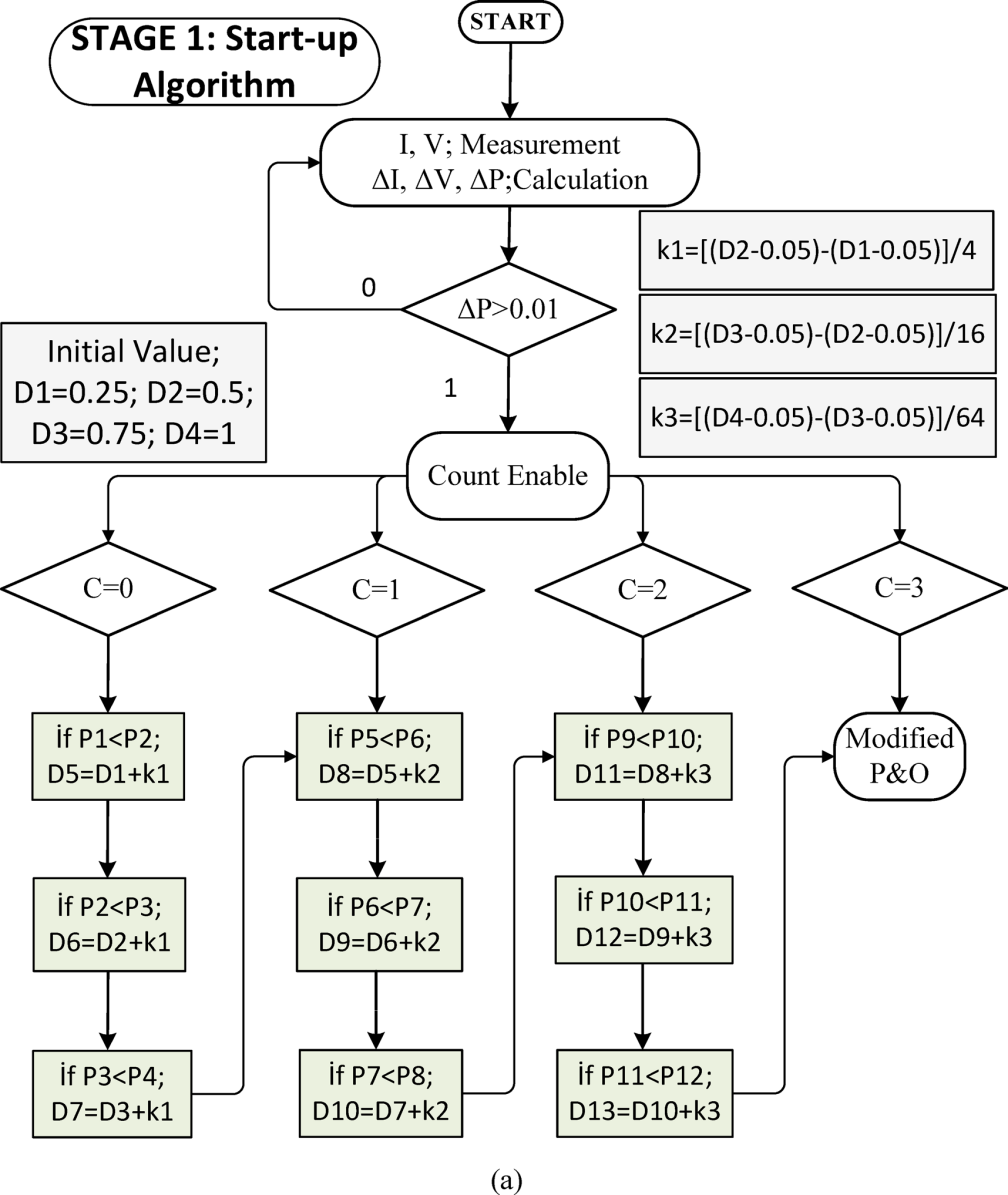

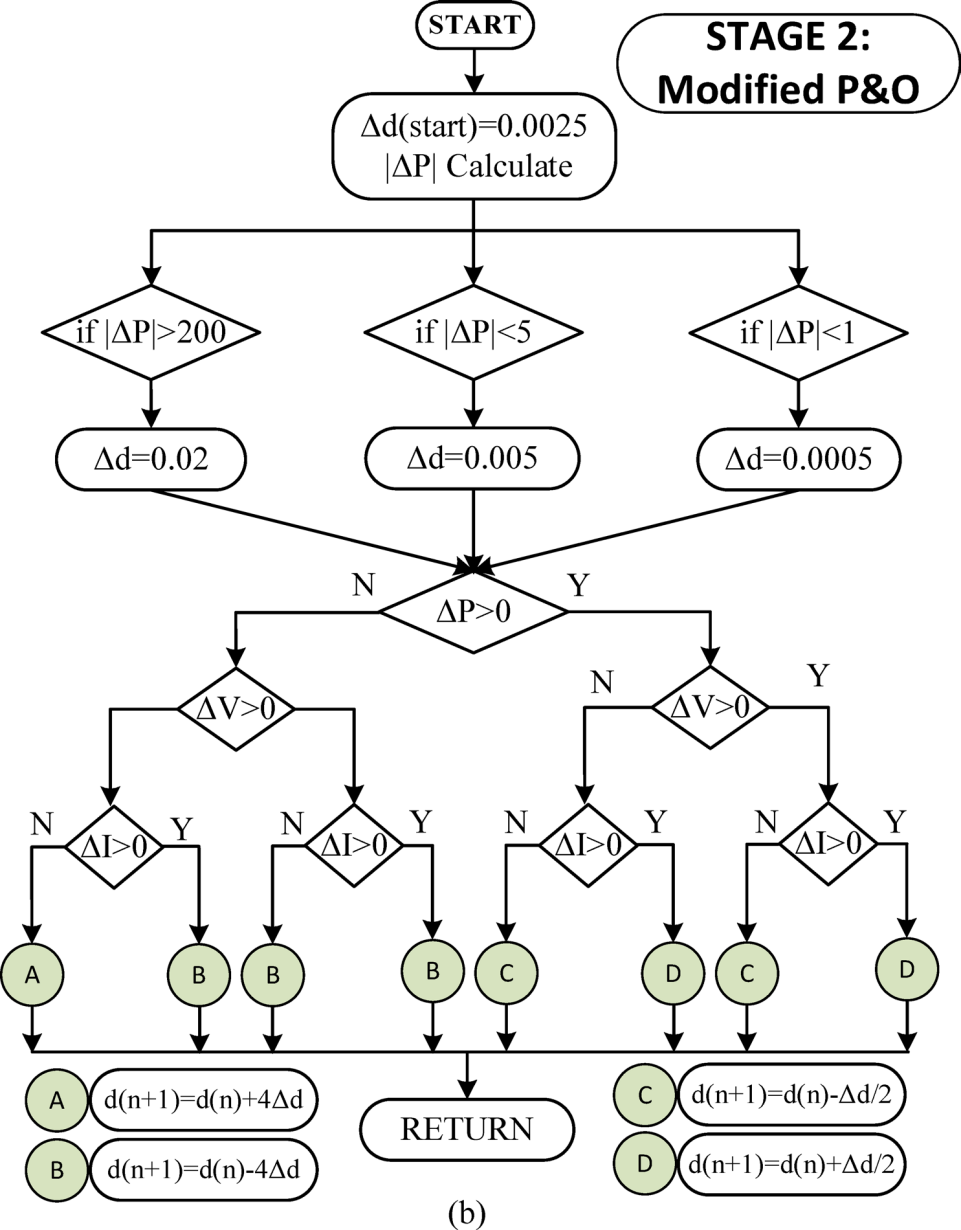
(**a**) Flowchart of starting process algorithm (**b**) Flowchart of modified P&O algorithm.

The flowchart of the MPPT algorithm to be applied during the start-up phase is shown in Fig. 3(a). When the count value reaches 3, it indicates that the maximum power is approached, and the last duty cycle, shown as D13 in Fig. 3(a), is transferred as the first duty cycle for the second stage.

22 $$\:\varDelta\:d=\left\{\begin{array}{c}0.02,\:\:\:\:\:\:\:\:\:\:\:\:if\:\varDelta\:P>200\\\:0.005,\:\:\:\:\:\:\:\:\:\:\:\:\:\:if\:\:\varDelta\:P<5\\\:0.0005,\:\:\:\:\:\:\:\:\:\:\:\:if\:\varDelta\:P<1\\\:0.0025,\:\:\:\:\:\:\:\:\:\:\:\:otherwise\end{array}\right..$$

In the traditional P&O method, Δ*d* is taken as a constant. Therefore, the traditional algorithm cannot adapt to transient conditions and also causes power fluctuations in the steady state. In the proposed algorithm, the maximum power is approached very quickly with the algorithm applied at the start-up. In the following cases, the second level M-P&O algorithm is run. In the second level M-P&O algorithm, the change in current is also observed in addition to the voltage value. Thus, it provides a fast response to transient conditions and power changes. The Δ value is determined with different values ​​for five different power change conditions. Thus, both the response to the transient condition is accelerated and the power fluctuations in the steady state are brought to very low levels.

In the second stage, the MPPT algorithm used in references^[[12] and [37]]^ has been updated. To improve the performance during the transition phase, a new Δ*d* duty cycle change rate has been determined based on the $\:\varDelta\:P$ variation seen in Eq. (22) at the start and in each cycle. The values of $\:\varDelta\:d$ used are shown in Eq. (22). Additionally, in the modified P&O algorithm, task period changes of $\:4\varDelta\:d\:and\:\varDelta\:d/2$ have been applied. Thus, very low power fluctuations have been achieved in the steady state. When the two-stage MPPT algorithm is applied sequentially, it quickly approaches the maximum power during the startup phase and maintains very low fluctuations in the steady state. The MPPT algorithm applied in the second stage is shown in Fig. 3(b).

## Simulation model and results

The proposed MPPT algorithm, designed to obtain maximum power from the PEMFC, has been tested using MATLAB/Simulink blocks. The parameters of the PEMFC used in the simulation are provided in Table 1, while the parameters of the boost converter are given in Table 2.

**Table 1:** Pa*r*ameters of PEMFC.

Parameter	Value	Unit
T	343	*K*
A	232	*cm* ^*2*^
N_FC_	35	
$\:{\varvec{\xi\:}}_{1}$	-0.944	
$\:{\varvec{\xi\:}}_{2}$	0.00354	
$\:{\varvec{\xi\:}}_{3}$	7.8 × 10^− 8^	
$\:\:{\varvec{\xi\:}}_{4}$	-1.96 × 10^− 4^	
I_lim_	2	*Acm* ^*− 2*^
$\:{\varvec{t}}_{\varvec{M}}$	0.0178	*cm*
$\:{\mathcal{k}}_{\varvec{H}2}$	4.22 × 10^− 5^	*kmol (atms)* ^*−1*^
$\:{\mathcal{k}}_{\varvec{O}2}$	2.11 × 10^− 5^	*kmol (atms)* ^*−1*^
$\:{\varvec{q}}_{{\varvec{H}}_{2}}$	10 × 10^− 5^	*kmol s* ^*− 1*^
$\:{\varvec{q}}_{{\varvec{O}}_{2}}$	5 × 10^− 5^	*kmol s* ^*− 1*^
$\:{\varvec{k}}_{\varvec{r}}$	9.07 × 10^− 8^	*kmol (sA)* ^*−1*^
*R*	8.31447	*J (kmol K)* ^*−1*^
$\:\varvec{F}$	96,484,600	*C kmol* ^*− 1*^

**Fig. 4:**
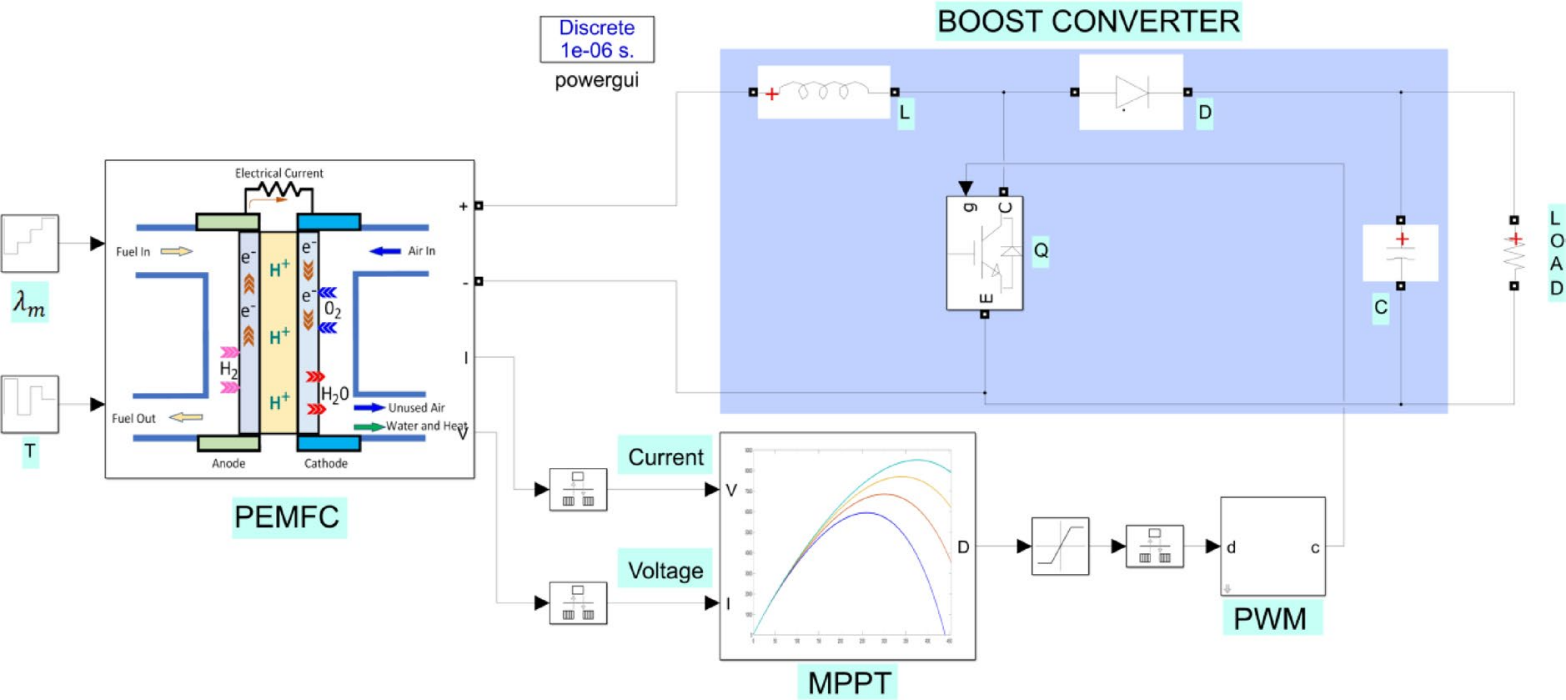
MATLAB simulation model of PEMFC power system.

A boost-type DC-DC converter has been used at the output of the PEMFC. The duty cycle generated by the MPPT algorithm is used for switching the DC-DC converter. This ensures that the switching occurs with the maximum power value transferred to the load. The simulation sampling time is 1 µs, while the switching frequency of the boost converter is 10 kHz. The MATLAB/Simulink simulation is shown in Fig. 4.

When the Q switch in the boost converter is on, the inductance voltage can be written as shown in Eq. (23). The duration that the switch Q stays in transmission can be written as $\:\varDelta\:t$, *DT*_*s*_. Here, *D* denotes the duty cycle of the switch Q, and *T*_*s*_ denotes the total time that the switch stays in the transmission and cut off, that is, the switching period. The duration in which the switch Q is cut-off can be written as *(1-D)T*_*s*_. In Eq. (24), the change in the current in the *L* inductance in the cut-off state of the Q switch is seen.

23 $$\:{V}_{s}\left(t\right)=L\frac{{dI}_{L}}{dt}\:and\:\frac{{\varDelta\:I}_{L}}{\varDelta\:t}=\frac{{V}_{s}}{L}$$

24 $$\:{\varDelta\:I}_{L}=\frac{{V}_{s}-{V}_{0}}{L}(1-D){T}_{s}\:$$.

The sum of the inductor current will be zero in the open and closed states of the switch, this situation is seen in Eq. (25).

25 $$\:\frac{{V}_{s}}{L}D{T}_{s}+\frac{{V}_{s}-{V}_{0}}{L}\left(1-D\right){T}_{s}=0$$.

Using Eq. (25), the relationship between the boost converter input and output voltages is be obtained as seen in Eq. (26).

26 $$\:\frac{{V}_{0}}{{V}_{s}}=\frac{1}{1-D}$$.

The minimum value that the inductance must have to operate in continuous current mode is given in Eq. (27)^38^.

27 $$\:{L}_{min}=\frac{{(1-D)}^{2}DR}{2{f}_{s}}$$.

In order for the output voltage to be within the desired ripple limits, the minimum value of the capacitance is calculated as follows.

28 $$\:{C}_{min}=\frac{D}{R{V}_{r}{f}_{s}}$$.

**Table 2:** Pa*r*ameters of boost converter.

Parameter	Value	Unit
L	2.9	*mH*
C	1500	*µF*
R	5	*Ω*
f_s_	10	*kHz*

**Fig. 5:**
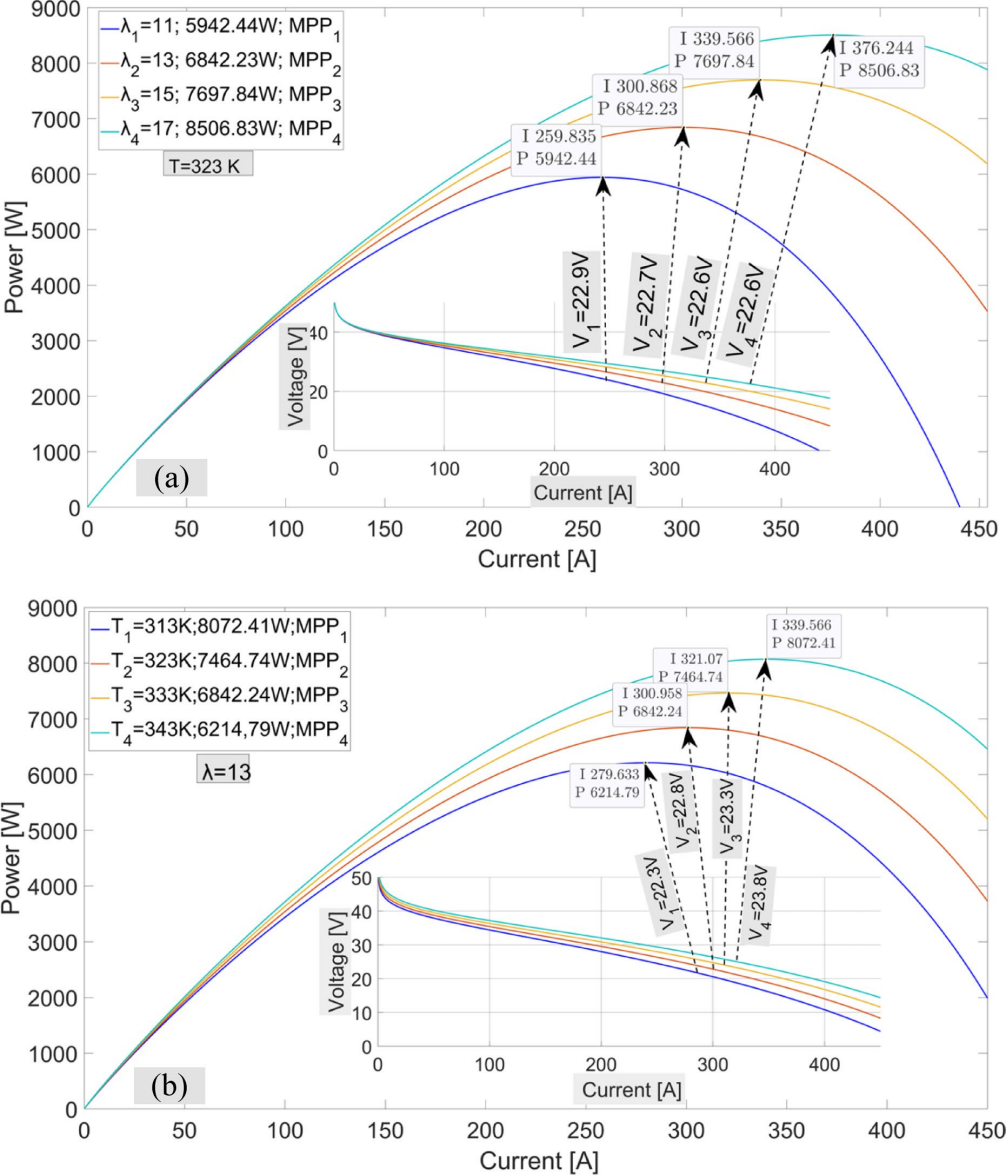
I-P graphs of the PEMFC power system. (**a**) Different $\:{\lambda\:}_{m}$ values (**b**) Different temperature values

**Fig. 6:**
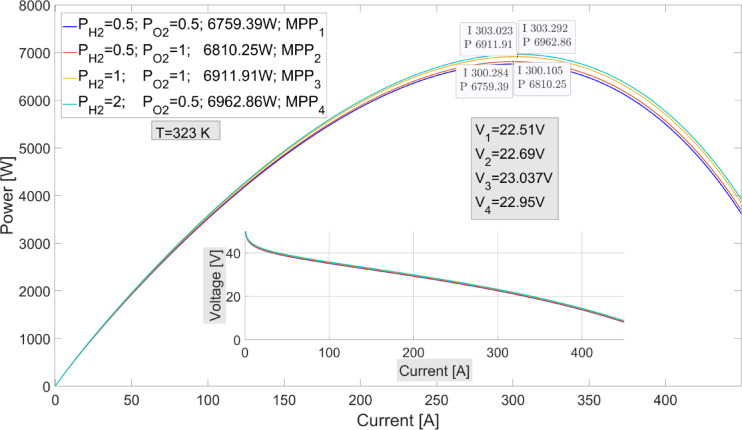
I-P graphs obtained with different oxygen and hydrogen pressures in PEMFC power system.

The membrane water content $\:({\lambda\:}_{m}$) and temperature variations of the fuel cell affect the maximum power values that can be obtained from the PEMFC. In Fig. 5(a), the maximum power values at a fixed temperature of 323 K and varying $\:{\lambda\:}_{m}$ values are shown. When examining the I-P graph, it can be observed that the current value changes significantly with variations in $\:{\lambda\:}_{m}\:$at the maximum power points. In contrast, the rate of change of the voltage is lower. When Fig. 5(b) is examined, the maximum power values at different temperatures are shown with a fixed $\:{\lambda\:}_{m}=13$. As seen in Fig. 5(b), the rate of change of current at maximum power points at different temperatures is higher than the voltage. In Fig. 6, the maximum power values ​​and the voltage values ​​at the moment of maximum power are shown at different oxygen and hydrogen pressure values ​​at the fixed value of $\:{\lambda\:}_{m}=13$.

The I-P graphs shown in Figs. 5 and 6 clearly demonstrate the necessity of using MPPT algorithms in PEMFC systems, as it is essential to find the current value that leads to the maximum power on the I-P curve. The P&O MPPT algorithm has been used in the simulation to demonstrate the performance of the proposed algorithm. The most important factor determining the performance of the P&O method is the change in the duty cycle value. In the simulation, results are shown for two different task periods: $\:\varDelta\:d=0.001\:\:$and $\:\varDelta\:d=0.0005$. In recent years, optimization-based MPPT algorithms such as PSO, CSA, and GA-based MPPT algorithms^39^ have also been used in the simulation.

**Fig. 7:**
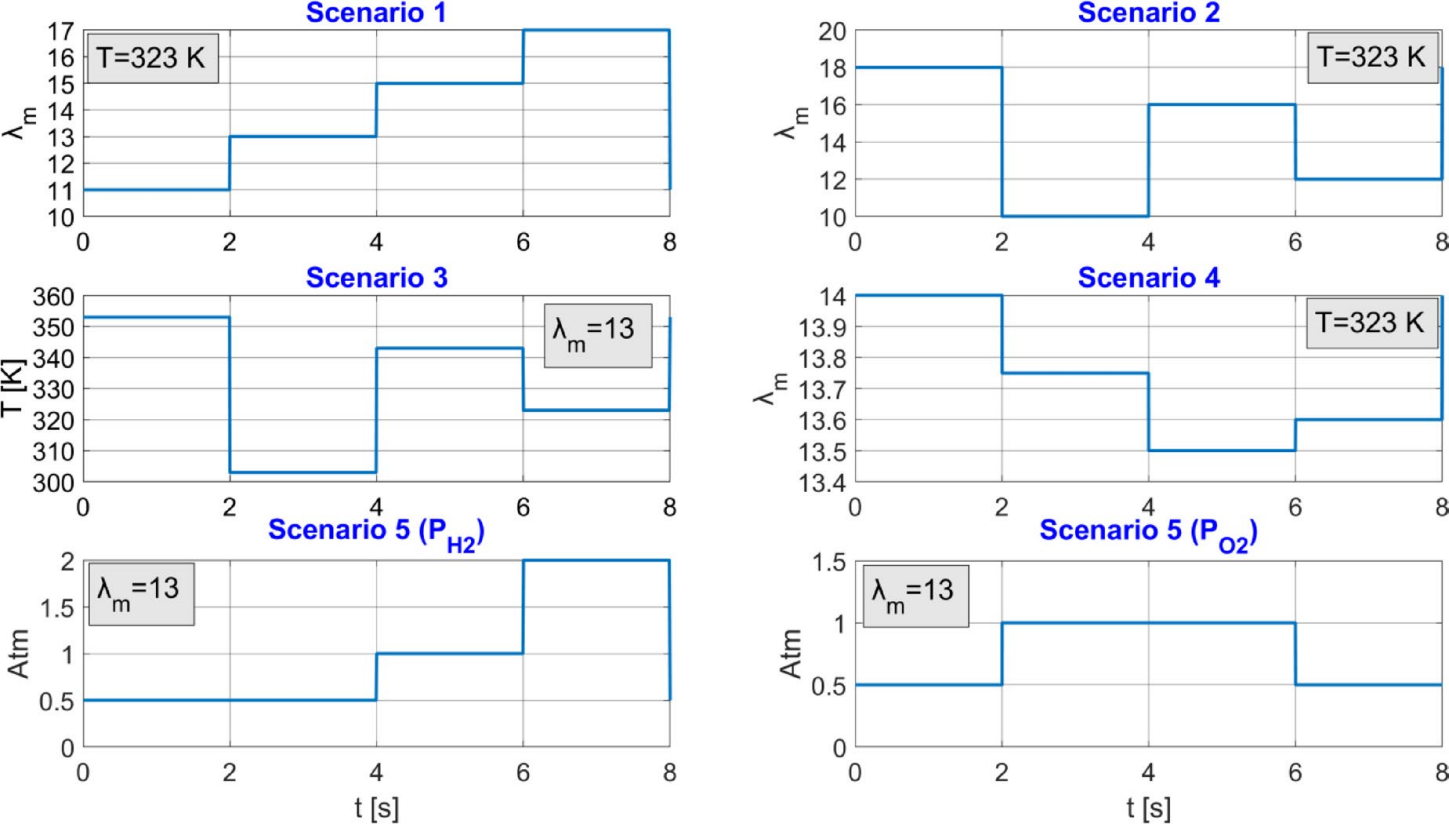
Five different scenarios created with temperature, $\:{\lambda\:}_{m}$, oxygen pressure, and hydrogen pressure variations in the PEMFC system

To demonstrate the success of the proposed algorithm, five different scenarios have been created, as shown in Fig. 7. In Scenario 1, the $\:{\lambda\:}_{m}$ values are gradually increased to 11, 13, 15, and 17 under a fixed temperature of 323 K. In Scenario 2, to observe the effect of large variations in $\:{\lambda\:}_{m}$, the values 18, 10, 16, and 12 are applied under a fixed temperature of 323 K. In Scenario 3, to observe the effect of temperature variations, the temperature is applied sequentially as 353, 303, 343, and 323 K, with $\:{\lambda\:}_{m}$=13 fixed. In Scenario 4, to observe the effect of small changes in $\:{\lambda\:}_{m}$, the values 14, 13.75, 13.5, and 13.6 are applied sequentially under a fixed temperature of 323 K. In Scenario 5, different oxygen and hydrogen pressures seen in Fig. 7 were applied to the system at constant $\:{\lambda\:}_{m}$=13 value.

**Fig. 8:**
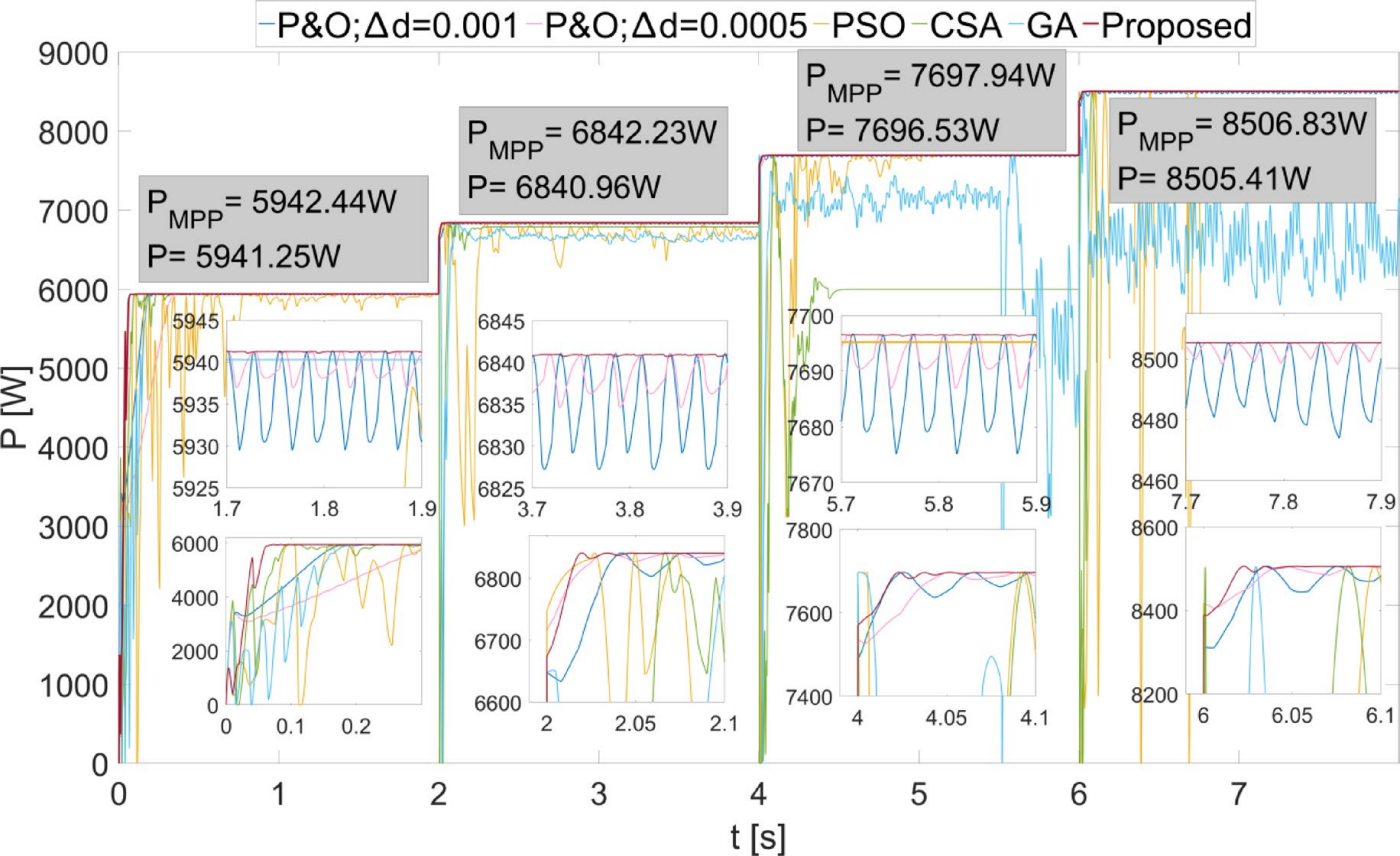
Power values obtained from PEMFC using different methods were obtained by applying Scenario 1.

**Fig. 9:**
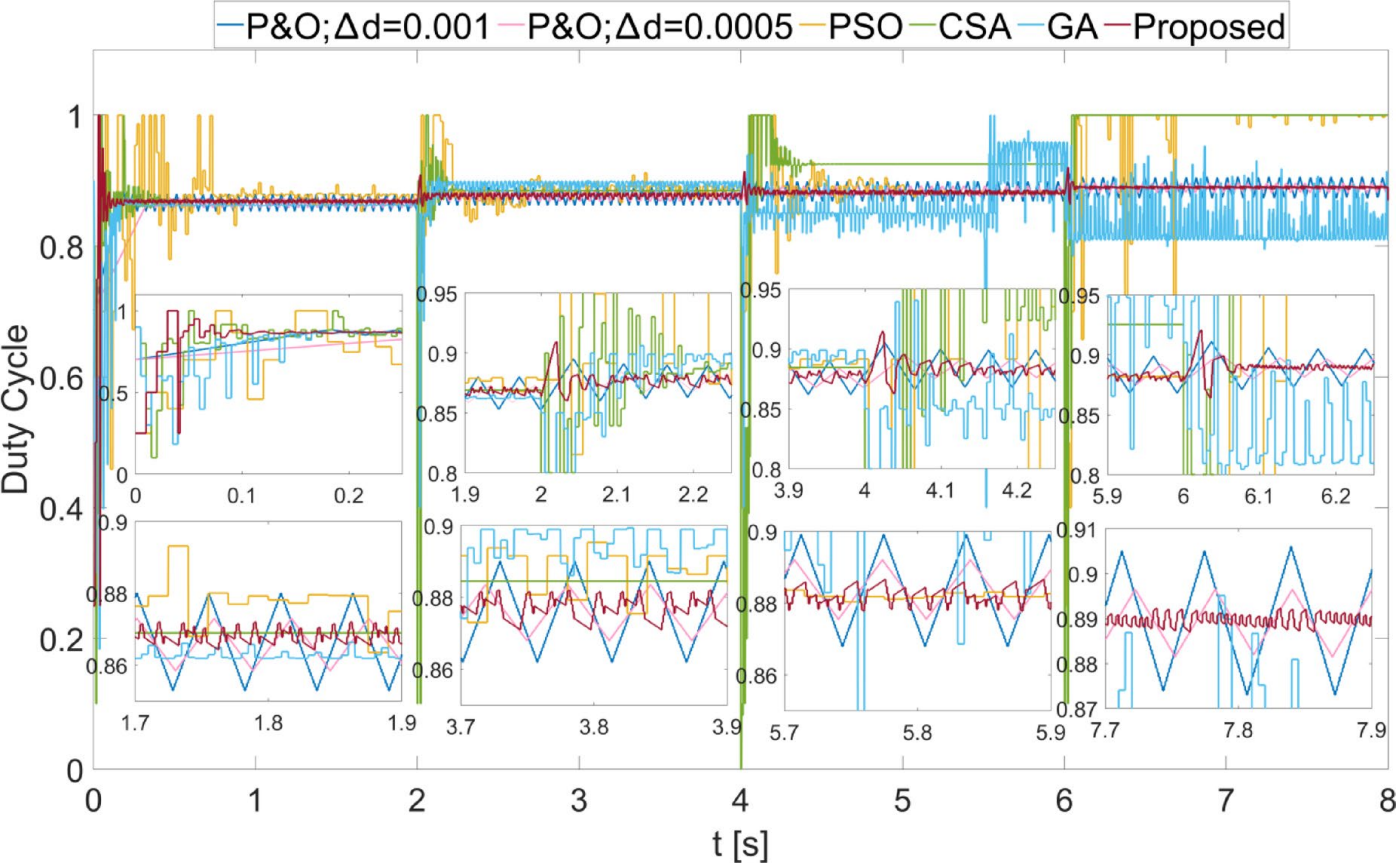
Duty cycles obtained with different methods when Scenario 1 was applied to the PEMFC system.

The power values obtained from the PEMFC system using Scenario 1 are shown in Fig. 8. In Fig. 8, two different duty cycle change values, : $\:\varDelta\:d=0.001\:\:$and $\:\varDelta\:d=0.0005$, are determined for the P&O method. Results obtained using the PSO, CSA, and GA optimization-based MPPT algorithms, along with the proposed algorithm, are also shown. It is known that when $\:\varDelta\:d$ is low in the P&O algorithm, power oscillations in the steady state will decrease, but the system will take longer to reach the new power value after a change. In Fig. 8, the results show that the power oscillations are lower when $\:\varDelta\:d=0.0005$, but the system reaches the steady state more slowly at the start-up stage. The PSO algorithm reaches the steady state after 2 s, CSA after 0.3 s, and GA after 0.2 s. The proposed method, however, reaches the steady state after 0.1 s. When $\:{\lambda\:}_{m}$ changes, the power values that can be obtained from the PEMFC also change. In each case, the proposed method reaches steady state more quickly. In all values of $\:{\lambda\:}_{m}$, the proposed method has successfully achieved the highest power output. Additionally, the power fluctuations in the steady state, based on $\:{\lambda\:}_{m}$ values, are very low with the proposed method, at 0.2 W, 0.35 W, 0.2 W, and 0.15 W, respectively. The power and efficiency values obtained with the proposed method are as follows: 5941.25 W; 99.98%, 6840.96 W; 99.98%, 7696.53 W; 99.98%, and 8505.41 W; 99.98%. Figure 9 shows the duty cycles obtained using the P&O, PSO, CSA, GA, and proposed methods when Scenario 1 is applied. At the moment the system starts, the first stage of the proposed method is activated. When the power variation decreases, the system continues working with the second stage. The speed of reaching steady state in the first stage is clearly visible from the change in duty cycles. In Fig. 9, the fast response of the duty cycle generated by the proposed method in the $\:{\lambda\:}_{m}\:$variations is seen as a significant advantage. The PSO method struggles to reach steady state. The duty cycles obtained with GA and CSA methods do not result in maximum power. The duty cycle generated by the proposed method in steady state, while varying depending on the power change, is influenced by the change in $\:\varDelta\:d$. The high performance of the modified P&O algorithm in both transient and steady states is clearly observed in Fig. 9.

**Fig. 10:**
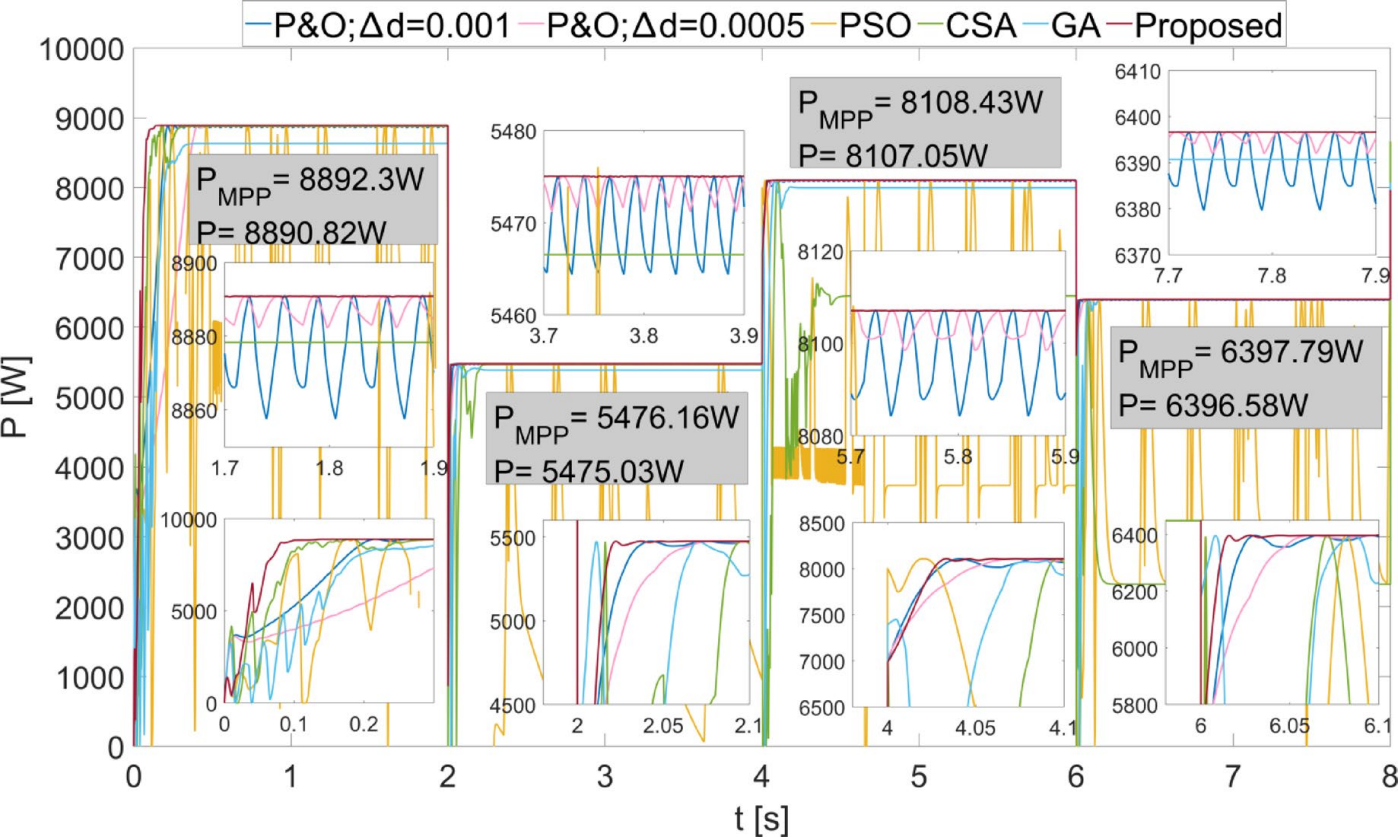
Power values obtained from the PEMFC using different methods by applying Scenario 2.

In Fig. 10, the power values obtained as a result of applying scenario 2 to the PEMFC are shown. During the initial energization, the proposed algorithm was the fastest to reach steady state. Additionally, with the proposed algorithm, the power obtained was the fastest to reach steady state as $\:{\lambda\:}_{m}$ changes. The proposed algorithm successfully achieved the highest power with the least fluctuation across all regions. The power fluctuations with the proposed method were observed to be very low, with values of 0.2 W, 0.25 W, 0.1 W, and 0.12 W according to $\:{\lambda\:}_{m}$ values. The power and efficiency obtained with the proposed method are 8892.82 W; 99.98%, 5475.03 W; 99.98%, 8107.05 W; 99.98%, and 6396.58 W; 99.98%, respectively.

**Fig. 11:**
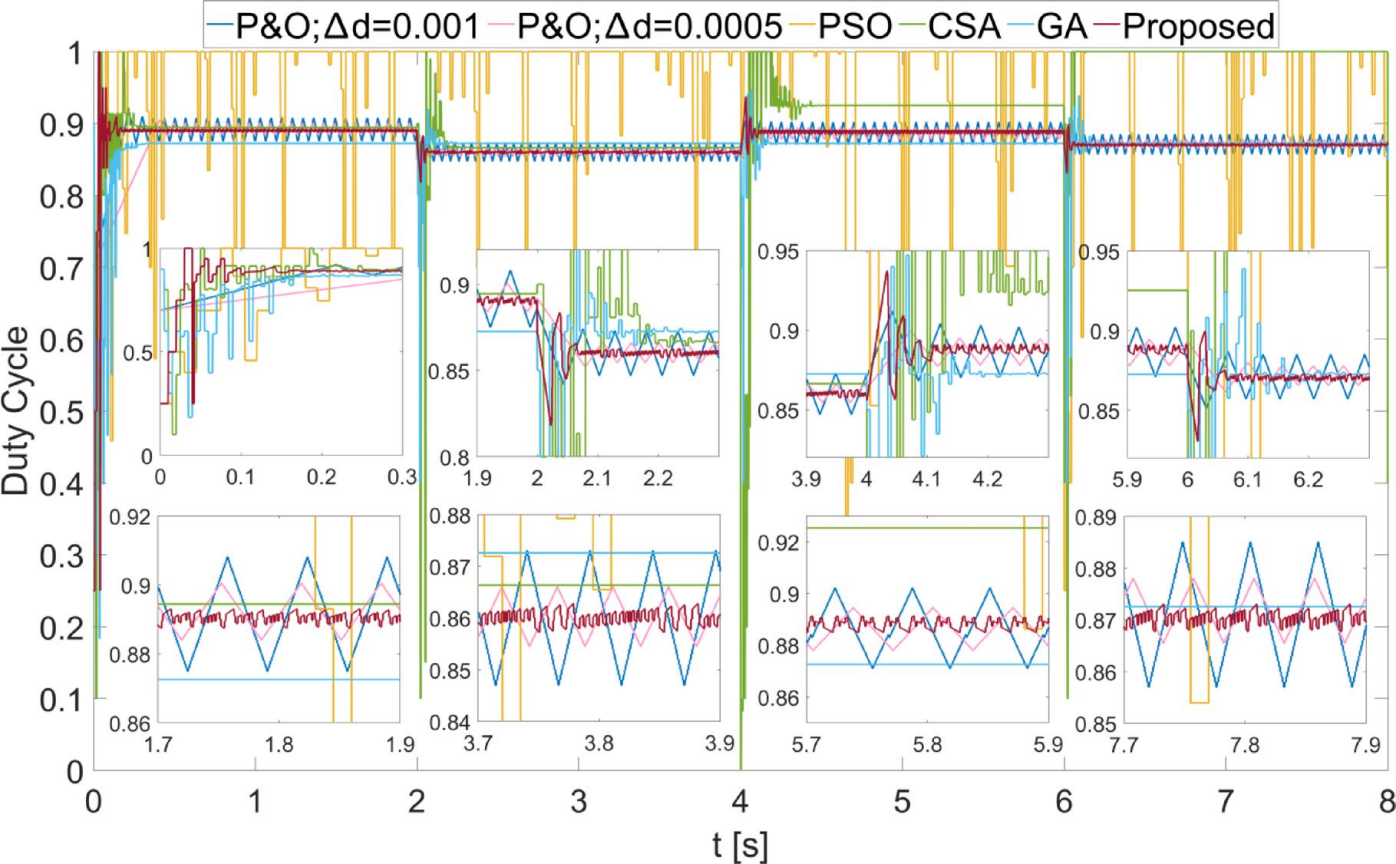
Duty cycles obtained with different methods when Scenario 2 is applied to the PEMFC.

In Fig. 11, the duty cycles obtained when scenario 2 was applied to the PEMFC are shown. It is evident from the duty cycle changes that the proposed method reaches steady state very quickly in the $\:{\lambda\:}_{m}$ variations. The PSO method never reached steady state and produced a highly unstable output. In the P&O method, when the $\:\varDelta\:d$ value was high, the duty cycle change was large, and consequently, the power fluctuations were high. The proposed method achieved a duty cycle with minimal change by adjusting the duty cycle according to the magnitude of the power changes. Thus, the power fluctuations were minimized.

**Fig. 12:**
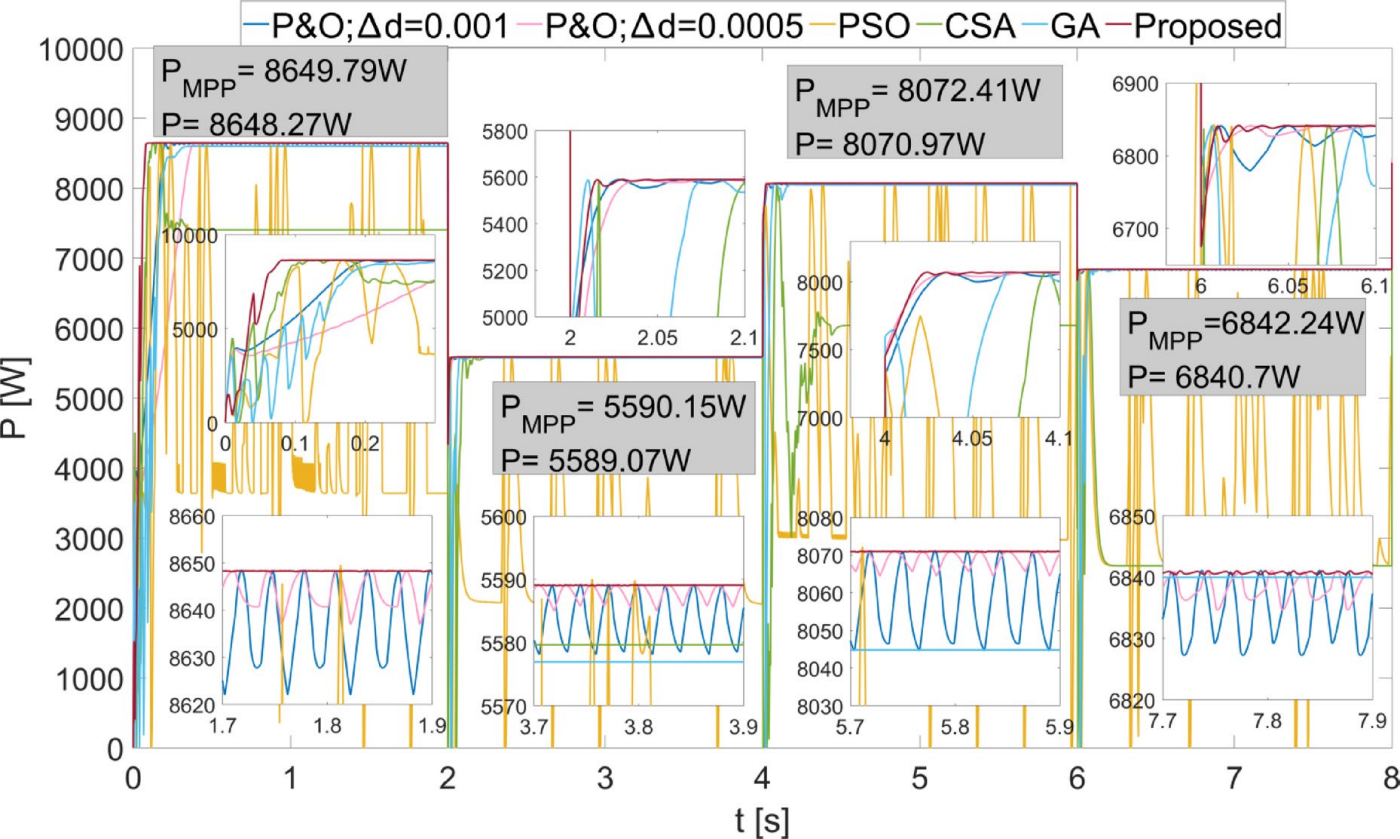
Power values obtained from PEMFC using different methods with Scenario 3.

**Fig. 13:**
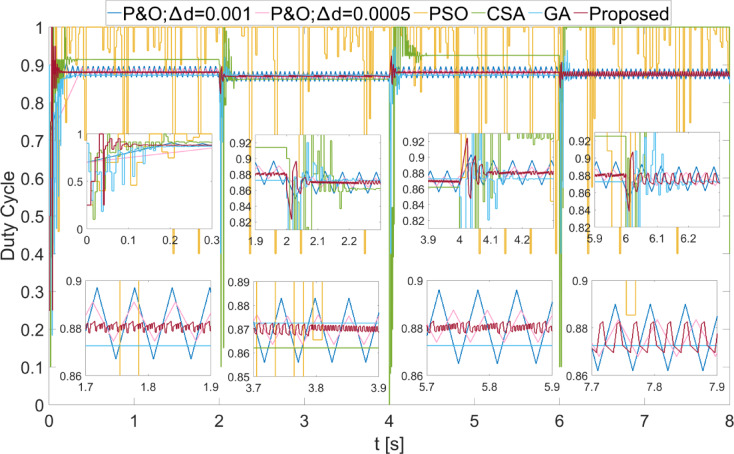
Duty cycles obtained with different methods when Scenario 3 is applied to the PEMFC.

In Fig. 12, the power values obtained for different temperature values in the PEMFC system operating under a fixed $\:{\lambda\:}_{m}\:\:$in Scenario 3 are shown. As in the other two scenarios, the proposed algorithm reaches the steady state the fastest after start-up. In steady operation, the power fluctuations are at a very low level for all conditions. The efficiency of the proposed method in steady state has been obtained as 99.98% for all different temperature values. The duty cycles obtained using P&O, PSO, CSA, GA, and the proposed method in Scenario 3 are shown in Fig. 13.

**Fig. 14:**
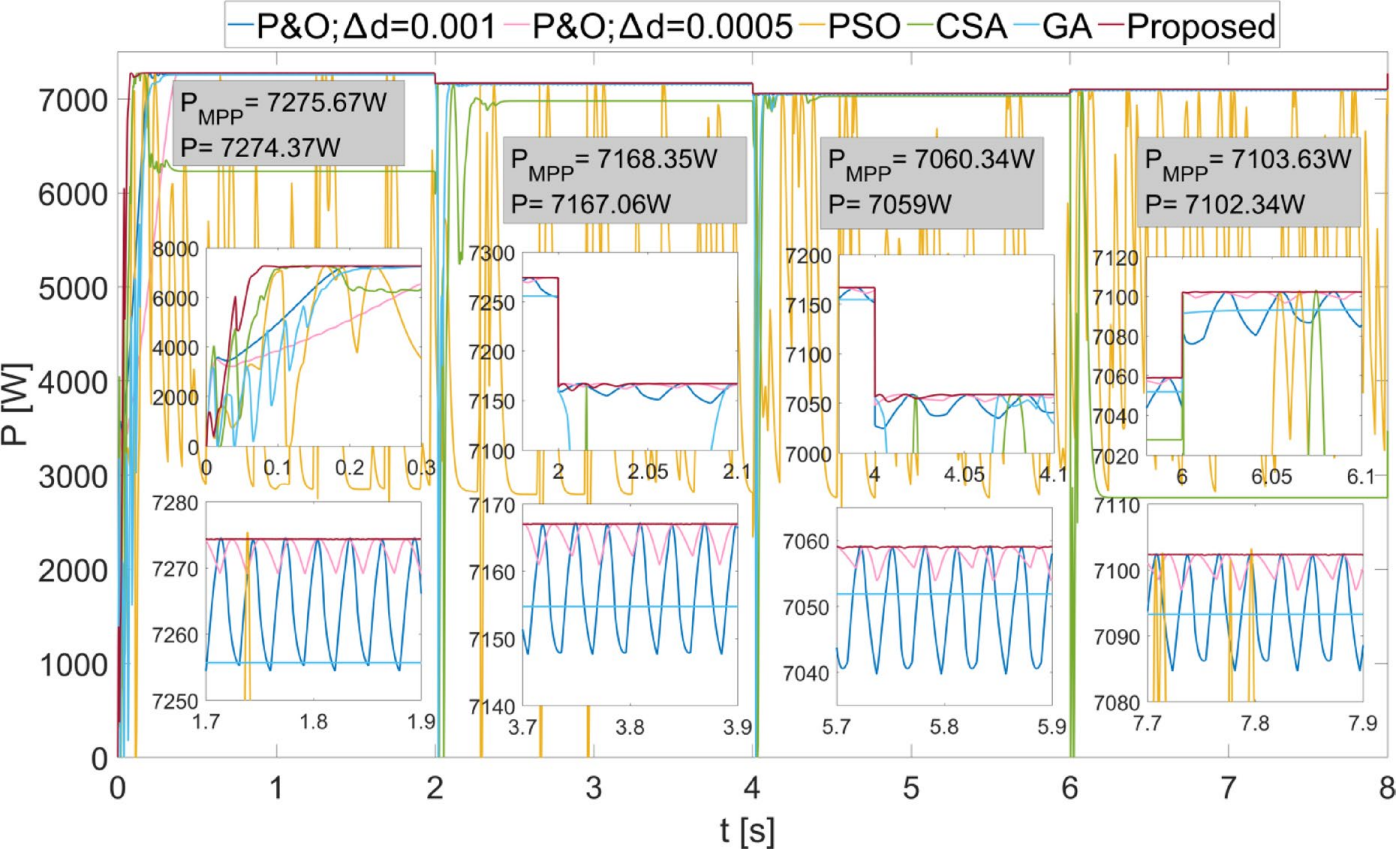
Powers obtained with different methods from the PEMFC when Scenario 4 is applied.

**Fig. 15:**
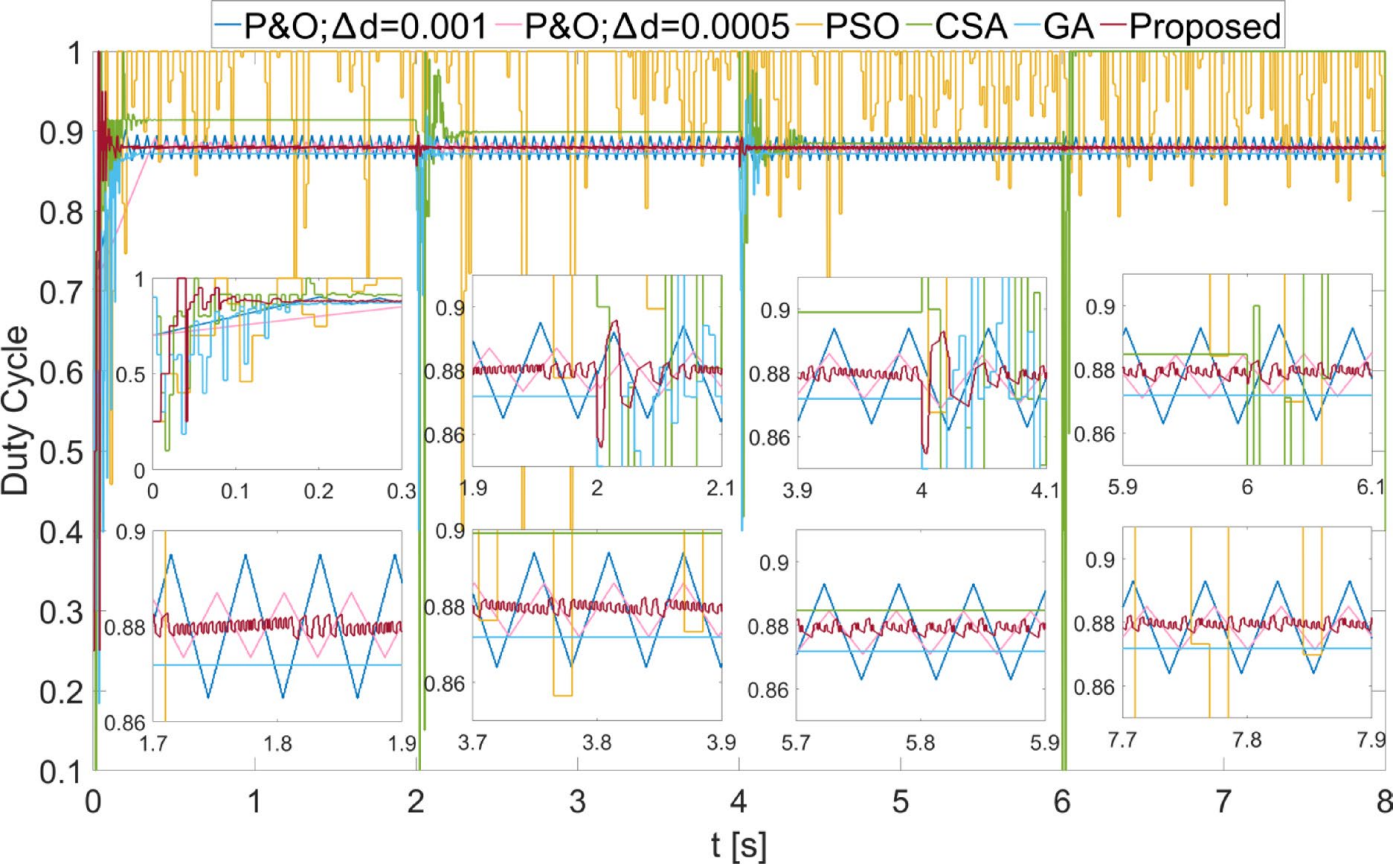
Duty cycles generated by different methods when Scenario 4 is applied to the PEMFC.

Scenario 4 was created to investigate the performance of MPPT algorithms under small variations. The success of the proposed method under small variations has been repeated, as seen in Fig. 14. The efficiency of the proposed MPPT algorithm has been obtained as 99.98% for all different values. The efficiencies of other methods have been obtained as lower. The power fluctuations obtained using the proposed method in steady state have occurred as 0.1 W, 0.1 W, 0.25 W, and 0.15 W for different values, respectively. The duty cycles obtained in Scenario 4 are shown in Fig. 15. Due to the low variation in power changes, the variation in the duty cycle obtained using the proposed method has also been low.

**Fig. 16:**
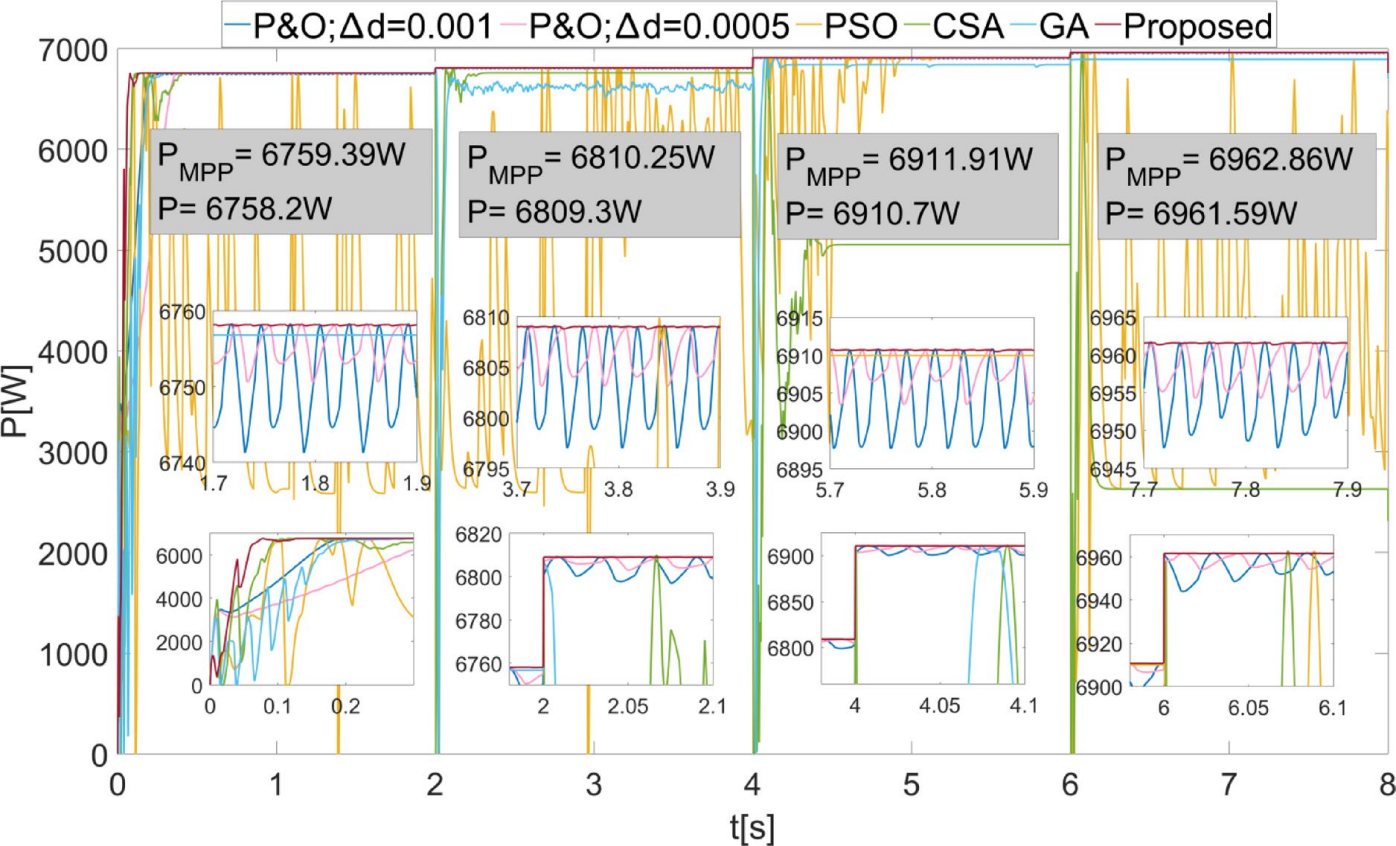
Powers obtained with different methods from the PEMFC when Scenario 5 is applied.

**Fig. 17:**
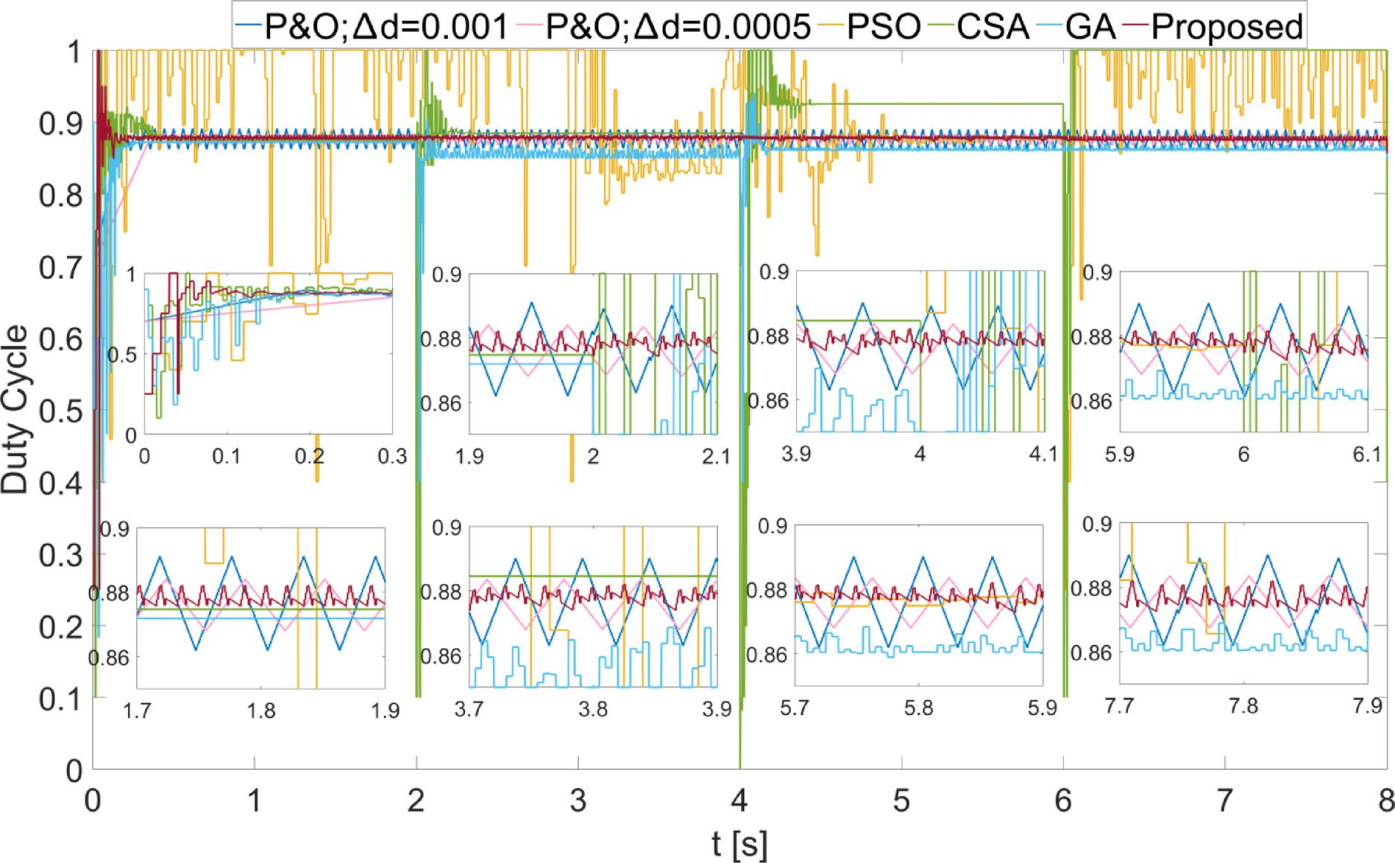
Duty cycles generated by different methods when Scenario 5 is applied to the PEMFC.

The success of the proposed method under small power variations while operating under different pressure values ​​has been repeated, as seen in Fig. 16. The efficiency of the proposed MPPT algorithm has been obtained as 99.98% for all different values. The power fluctuations obtained using the proposed method in steady state have occurred as 0.15 W, 0.1 W, 0.2 W, and 0.25 W for different values, respectively. The duty cycles obtained in Scenario 5 are shown in Fig. 17.

**Table 3:** Tracking speed, power fluctuation and efficiency in five different scenarios for P&O (∆d = 0.001), P&O (∆d = 0.0005), and proposed Method.

		*P*&O $\:\varDelta\:\varvec{d}=0.001$			*P*&O $\:\varDelta\:\varvec{d}=0.0005$			Proposed Method		
		T.S.	*P*.F.	Eff.	T.S.	*P*.F.	Eff.	T.S.	*P*.F.	Eff.
**Scenario1**	0–2 s	0.18	11.63	99.85	0.328	4.13	99.94	0.061	0.2	99.98
	2–4 s	2.042	13.61	99.88	2.034	6.04	99.93	2.019	0.35	99.98
	4–6 s	4.026	20.65	99.85	4.05	9.11	99.92	4.023	0.2	99.98
	6–8 s	6.036	26.55	99.8	6.045	4.73	99.94	6.022	0.15	99.98
**Scenario2**	0–2 s	0.216	24.55	99.81	0.39	7.41	99.93	0.08	0.2	99.98
	2–4 s	2.043	10.1	99.87	2.07	3.45	99.94	2.021	0.25	99.98
	4–6 s	4.04	22.6	99.84	4.065	8.18	99.93	4.03	0.1	99.98
	6–8 s	6.03	16.4	99.86	6.057	4.32	99.95	6.01	0.12	99.98
**Scenario3**	0–2 s	0.216	25.39	99.84	0.378	10.63	99.92	0.09	0.15	99.98
	2–4 s	2.027	10.13	99.87	2.043	3.67	99.95	2.016	0.05	99.98
	4–6 s	4.035	26.17	99.81	4.036	5.67	99.94	4.02	0.15	99.98
	6–8 s	6.0126	13.47	99.88	6.028	5.9	99.93	6.01	0.6	99.98
**Scenario4**	0–2 s	0.1966	19.6	99.83	0.3669	4.94	99.94	0.077	0.1	99.98
	2–4 s	2.0007	19.2	99.83	2.0007	6.02	99.94	2.0007	0.1	99.98
	4–6 s	4.0007	18.74	99.84	4.0007	4.9	99.95	4.0007	0.3	99.98
	6–8 s	6.0005	17.6	99.85	6.0005	7	99.95	6.0005	0.15	99.98
**Scenario5**	0–2 s	0.1824	16.42	99.86	0.3463	7.42	99.92	0.077	0.15	99.98
	2–4 s	2.009	11.82	99.89	2.009	5.66	99.93	2.0007	0.1	99.98
	4–6 s	4.0064	12.8	99.88	4.024	5.9	99.93	4.0007	0.2	99.98
	6–8 s	6.0008	13.67	99.88	6.01	7.06	99.92	6.0005	0.25	99.98
**T.S**: Tracking Speed (s) **P.F**: Power Fluctuation (W) **Eff**: Efficiency (%) (Steady-State)										

In order to see the superiority of the proposed method, the Tracking Speed, Power Fluctuation and Efficiency results of P&O ($\:\varDelta\:d=0.001$), P&O $\:(\varDelta\:d=0.0005$) and proposed methods obtained with five different scenarios are given in Table 3. Accordingly, in all scenarios and different regions, the power fluctuations in the steady state were much lower in the proposed method. In all cases, the efficiency of the proposed method was higher than the other methods and was obtained as 99.98%.

The time from the start moment to reaching the maximum power was obtained much shorter in the proposed method. In the case of $\:\varDelta\:d=0.0005$, although the efficiency of the P&O method increased due to the decrease in power fluctuations, the time to reach the steady state decreased.

In the results obtained for different temperature and$\:\lambda\:m$ values in five different scenarios, the proposed algorithm’s ability to generate power with low fluctuation and operate with 99.98% efficiency in steady-state operation has emerged as a significant advantage. In transient states, the algorithm’s ability to quickly reach steady-state operation has been an important success criterion. The simple structure of the algorithm, compared to optimization algorithms and intelligent algorithms, will provide a significant advantage in practical applications.

## Conclusion

Renewable energy sources, such as solar and wind energy, are highly dependent on atmospheric conditions. Therefore, the continuity of energy supply is a significant challenge in energy production systems using these sources. PEMFC systems are gaining importance as renewable energy solutions due to their low emissions and high efficiency. Their low operating temperature and high power density are considered important advantages. Additionally, the ability to store hydrogen eliminates the problem of energy continuity. The power produced by PEMFC systems reaches a maximum at a specific current value. Therefore, MPPT algorithms are employed to track this maximum power. In the literature, alongside traditional MPPT algorithms like P&O, optimization-based and intelligent methods are also used to maximize power output. However, optimization-based and intelligent methods tend to be complex and computationally intensive, making their implementation more challenging compared to traditional methods.

In this study, the P&O algorithm has been modified to develop a simple yet high-performance MPPT algorithm. The proposed algorithm consists of two stages. The first stage operates during the start-up period, and after reaching steady-state operation, the second stage runs continuously. To evaluate the proposed algorithm, a PEMFC power system was modeled in the MATLAB/Simulink environment. A boost converter was employed as the DC-DC converter, with the duty cycle generated by the MPPT algorithm applied to it. The proposed algorithm was compared with P&O, PSO, CSA, and GA algorithms. The P&O algorithm was tested with two different ∆d values. All algorithms were evaluated under five different scenarios with identical conditions and sampling times. In all scenarios, the proposed algorithm achieved very high efficiency (99.98%) during steady-state operation. Power fluctuations using the proposed algorithm in steady state ranged between 0.1 W and 0.6 W. In contrast, the traditional P&O algorithm exhibited much higher power fluctuations, which varied depending on the ∆*d* value. The optimization algorithms did not achieve consistent success across all scenarios, showing lower efficiencies compared to the proposed algorithm. Notably, the PSO-based MPPT algorithm performed poorly. The proposed algorithm responded very quickly to power changes, reaching steady-state operation in less than 0.1 s during startup.

In future studies, the Δd value of the proposed algorithm will be adjusted using fuzzy logic. This will enable the development of a more flexible MPPT algorithm that can better adapt to both small and large power changes, maintaining high performance during transient states.

## Data Availability

The datasets used and/or analysed during the current study available from the corresponding author on reasonable request.

## Abbreviations

PEMFC: Proton Exchange Membrane Fuel Cell
MPPT: Maximum Power Point Tracking
P&O: Perturb and Observe
InC: Incremental Conductance
PSO: Particle Swarm Optimization
CSA: Cuckoo Search Algorithm
GA: Genetic Algorithm
ANN: Artificial neural Networks
FL: Fuzzy Logic
M-P&O: Modified Perturb and Observe
$\:{\varvec{q}}_{{\varvec{H}}_{2}}$: Hydrogen flow rate
$\:{\varvec{q}}_{{\varvec{O}}_{2}}$: Oxygen flow rate
$\:{\varvec{P}}_{{\varvec{H}}_{2}}$: Hydrogen partial pressure
$\:{\varvec{P}}_{{\varvec{O}}_{2}}$: Oxygen partial pressure
$\:{\mathcal{k}}_{\varvec{H}2}$: Hydrogen valve constant
$\:{\mathcal{k}}_{\varvec{O}2}$: Oxygen valve constant
$\:{\varvec{M}}_{\varvec{H}2}$: Hydrogen molar mass
$\:{\varvec{M}}_{\varvec{O}2}$: Oxygen molar mass
R: Gas constant
*T*: Absolute temperature
$$\:{\varvec{V}}_{\varvec{a}\varvec{n}}$$: Anode volume
$\:{\varvec{q}}_{{\varvec{H}}_{2}}^{\varvec{i}\varvec{n}}$: Hydrogen inlet flow
$\:{\varvec{q}}_{{\varvec{H}}_{2}}^{\varvec{o}\varvec{u}\varvec{t}}\:\:$: Hydrogen outlet flow
$\:{\varvec{q}}_{{\varvec{H}}_{2}}^{\varvec{r}}$: Hydrogen flow entering the reaction
$\:{\varvec{N}}_{\varvec{F}\varvec{C}}$: Number of series-wound PEMFCs
$\:{\varvec{I}}_{\varvec{F}\varvec{C}}$: Current of the PEMFC
$$\:\varvec{F}$$: Faraday constant
$$\:{\varvec{k}}_{\varvec{r}}\:\:$$: Model constant
$\:{\varvec{E}}_{\varvec{n}\varvec{e}\varvec{r}\varvec{s}\varvec{t}}$: Thermodynamic equilibrium voltage
$\:{\varvec{V}}_{\varvec{a}\varvec{c}\varvec{t}}$: Activation voltage losses
$\:{\varvec{V}}_{\varvec{o}\varvec{h}\varvec{m}\varvec{i}\varvec{c}}$: Ohmic voltage loss
$\:{\varvec{V}}_{\varvec{c}\varvec{o}\varvec{n}}$: Concentration voltage loss
$$\:{\varvec{\xi\:}}_{1},\:{\varvec{\xi\:}}_{2},\:{\varvec{\xi\:}}_{3},\:{\varvec{\xi\:}}_{4}$$: Parametric coefficients
$$\:{\varvec{C}}_{{\varvec{O}}_{2}}$$: Oxygen concentration
$$\:{\varvec{r}}_{\varvec{M}}$$: Membrane resistance
$$\:{\varvec{t}}_{\varvec{M}}$$: Membrane thickness
A: Active area of the cell
$\:{\varvec{\lambda\:}}_{\varvec{m}}$: Average water activity
$$\:\varDelta\:\varvec{P}$$: Power change
$\:\varDelta\:\varvec{d}$: Duty cycle change
k: Duty cycle increase rate in Stage 1
$\:{\varvec{D}}_{\varvec{n}}$: Duty cycle in Stage 1
